# To Treat or Not to Treat? This Is the Question… About the Incidental Finding of Double Sinus of Valsalva Aneurysm in A 91-Year-Old Woman

**DOI:** 10.21470/1678-9741-2021-0403

**Published:** 2022

**Authors:** Luca Di Marco, Giuseppe Comentale, Matteo Bruno, Valerio Lanzillotti, Mauro Colletta, Vincenzo Russo, Davide Pacini, Gianni Casella

**Affiliations:** 1 Cardiac Surgery, IRCCS Azienda Ospedaliero-Universitaria di Bologna, S. Orsola Hospital, Bologna, Italy.; 2 Division of Cardiology, Maggiore Hospital, Bologna, Italy.

**Keywords:** Valsalva Sinus, Aneurysm, Coronary Artery

## Abstract

Sinus of Valsalva aneurysm is a very uncommon clinical finding and often requires emergency surgery due to its high risk of rupture. This educational text reports the case of a 91-year-old Italian women who was incidentally discovered to have a huge double aneurysm of the sinuses of Valsalva.

**Table:** Abbreviations, Acronyms & Symbols

CTA	= Computed tomography angiography
ECG	= Electrocardiogram
hsTnI	= High-sensitivity troponin I
SVA	= Sinus of Valsalva aneurysm

## INTRODUCTION

**Fig. 1:**
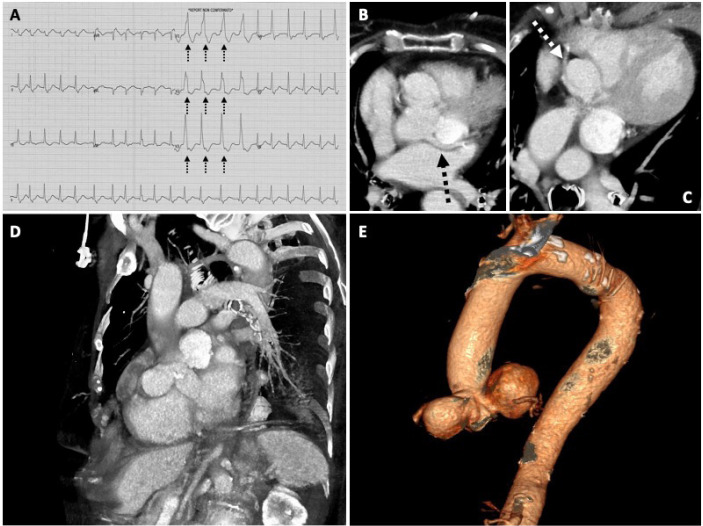
(A) Admission electrocardiogram showing anterolateral ST-segment depression (black arrows). Compression of left (B) and right (C) coronary artery by the two sinuses of Valsalva aneurysms (black and white arrow). Multiplanar (D) and 3D volume rendering (E) reconstruction of the aortic root showing the two sinuses of Valsalva aneurysms.

Sinus of Valsalva aneurysm is a very uncommon finding and often requires emergency surgery due to its high risk of rupture. This educational text reports the case of a 91-year-old Italian woman who was incidentally discovered to have a huge double aneurysm of the sinuses of Valsalva. The patient was admitted to the emergency department due to acute palpitation, chest pain and shortness of breath. Medical history revealed hypertension, dyslipidaemia and no other cardiovascular risk factors or previous event of cardiac arrhythmias. The admission electrocardiogram (ECG) showed the presence of atrial fibrillation with a pulse rate of 130 bpm, which was treated with intravenous infusions of amiodarone (bolus 300 mg/20 min and then continuous infusion of 900 mg/50 mL NaCl 0.9% at an infusion rate of 2 mL/h) that restored the normal sinus rhythm. Due to the anterolateral ST-segment depression (Figure 1A) and serum high-sensitivity troponin I levels (hsTnI) of 903 ng/L, the patient underwent a coronary angiogram. The right coronary artery was found to be free from stenosis, but during attempts to study the left coronary artery, an abnormal stagnation of contrast was noted inside the left sinus of Valsalva, therefore a computed tomography angiography (CTA) scan was performed in the suspicion of acute aortic dissection. The CT scan showed no signs of aortic dissection, but surprisingly revealed the presence of two huge and distinct dilatations of the left (5.5 × 5 cm) and right (4.7 ×4.2 cm) sinuses of Valsalva (Figure 1) that compressed the first segment of the left (Figure 1B, black arrow) and right (Figure 1C, white arrow) coronary artery. Basing on the CTA findings, the case was discussed by the Heart Team and based on the patient's age, incidental finding, and absence of urgent conditions requiring treatment, the Heart Team decided to address the patient to a conservative approach through medical therapy and cardiological follow-up.

### Questions

- What was the cause of the acute onset of atrial fibrillation in a patient with no history of cardiac arrythmias?
- If the electrocardiogram did not show any signs of myocardial ischemia, would you have performed a coronary angiogram or a CTA?
- Regarding the treatment options, what would be the surgical challenges?
- What was the main reason for choosing the medical treatment?

### Discussion of the Questions

The acute onset of atrial fibrillation in such an elderly patient with no previous history of cardiac arrythmias is usually related to high blood pressure rather than atrial enlargement related to mitral valve diseases (Question A).

One of the most crucial points of this case was the presence of electrocardiographic signs of myocardial ischemia. If the ECG did not show anterolateral ST-segment depression along with abnormal serum troponin levels, restoration of sinus rhythm after amiodarone infusion probably would have not prompt clinicians to perform a coronary angiogram or CTA scan and the real problem would be missed. Idiopathic atrial fibrillation, indeed, is a common cause of palpitation, chest pain and shortness of breath in elderly patients (Question B).

Due to the high risk of rupture, open surgery is usually the gold standard treatment. In this particular case, the patient should undergo a Bentall-De Bono procedure probably with a Cabrol technique to reimplant the coronary arteries. The sinus of Valsalva aneurysm has, in fact, moved the coronary ostia away from their natural position in relation to the aortic root, therefore the only way to reconnect them to the aorta would require the interposition of a prosthetic conduit. Considering the advanced age of the patient and her life expectancy, the procedure would have been very long and with a high risk of intra- and postoperative complications (Question C).

The choice to address the patient for medical treatment only was driven by a multifactorial analysis based on the patient's age, the occasional nature of the finding, the high perioperative risk, the absence of signs of imminent rupture and the good blood pressure control at home (Question D).

### Brief Consideration of the Reported Case

Sinus of Valsalva aneurysm (SVA) is an extremely rare finding, affecting about 0.09% of the general population^[1]^, and is usually an incidental report. When symptomatic, SVA usually present with signs of aortic rupture, angina (due to compression of the coronary artery), cardiac arrhythmias or with symptoms related to the compression of the neighbouring structures. A recent review showed that, as observed in this case, atrial fibrillation was the most reported arrhythmia (9%), while myocardial ischemia was present in only 5 cases among the 53 reported and was related to the high SVA dimensions^[1]^. However, the reported review showed that the oldest treated patients that could be found in the literature were 84 years old^[2]^. To the best of our knowledge, this could probably be the oldest reported patient in which a double sinus of Valsalva aneurysm remains asymptomatic until 91 years of age, therefore, considering the good blood pressure control obtained by patients at home with medical therapy only, the Heart Team decision was in favor of periodic follow-up. Unfortunately, we do not know the exact etiology of the double aneurysm, although we think it could be a congenital anomaly or, less likely, a consequence related either to hypertension or to a previous dissection limited to sinuses of Valsalva.

### Learning Points

- Even in the absence of signs of myocardial ischemia, atrial fibrillation may be the unusual presentation of evolving aortic root diseases, especially in elderly patients with hypertension.
- Due to the high risk of rupture, urgent open surgery is usually the gold standard treatment for SVA.
- SVA is usually an incidental finding and may be missed or undiagnosed in asymptomatic or even symptomatic patients.

**Table:** Authors’ Roles & Responsibilities

LDM	Substantial contributions to the conception or design of the work; or the acquisition, analysis, or interpretation of data for the work; drafting the work or revising it critically for important intellectual content; final approval of the version to be published
GC	Substantial contributions to the conception or design of the work; or the acquisition, analysis, or interpretation of data for the work; drafting the work or revising it critically for important intellectual content; final approval of the version to be published
MB	Substantial contributions to the conception or design of the work; or the acquisition, analysis, or interpretation of data for the work; drafting the work or revising it critically for important intellectual content; final approval of the version to be published
VL	Substantial contributions to the conception or design of the work; or the acquisition, analysis, or interpretation of data for the work; drafting the work or revising it critically for important intellectual content; final approval of the version to be published
MC	Substantial contributions to the conception or design of the work; or the acquisition, analysis, or interpretation of data for the work; drafting the work or revising it critically for important intellectual content; final approval of the version to be published
VR	Substantial contributions to the conception or design of the work; or the acquisition, analysis, or interpretation of data for the work; drafting the work or revising it critically for important intellectual content; final approval of the version to be published
DP	Substantial contributions to the conception or design of the work; or the acquisition, analysis, or interpretation of data for the work; drafting the work or revising it critically for important intellectual content; final approval of the version to be published
GC	Substantial contributions to the conception or design of the work; or the acquisition, analysis, or interpretation of data for the work; drafting the work or revising it critically for important intellectual content; final approval of the version to be published
